# CRISPR/Cas9-mediated *FeS-ELF3* inactivation overcomes heteromorphic self-incompatibility in common buckwheat

**DOI:** 10.3389/fpls.2026.1919002

**Published:** 2026-09-21

**Authors:** Artur Pinski, Magdalena Zaranek, Joanna Lusinska, Przemyslaw Kopec, Agnieszka Plazek, Meiliang Zhou, Alexander Betekhtin

**Affiliations:** 1 Institute of Biology, Biotechnology and Environmental Protection, Faculty of Natural Sciences, University of Silesia in Katowice, Katowice, Poland; 2 Department of Plant Cytology and Embryology, Institute of Botany, Faculty of Biology, Jagiellonian University in Kraków, Kraków, Poland; 3 The Franciszek Górski Institute of Plant Physiology, Polish Academy of Sciences, Kraków, Poland; 4 Department of Plant Breeding, Physiology and Seed Science, University of Agriculture, Kraków, Poland; 5 Institute of Crop Sciences, Chinese Academy of Agricultural Sciences, Beijing, China

**Keywords:** buckwheat, CRISPR/Cas9 system, *Fagopyrum esculentum*, heterostylous self-incompatibility, plant transformation

## Abstract

Common buckwheat (*Fagopyrum esculentum*) is a climate-resilient pseudocereal; however, its global adoption is constrained by the heteromorphic self-incompatibility associated with distyly. While the *S-locus early flowering 3* (*FeS-ELF3*) gene has been identified as a key regulator of self-compatibility, a stable genetic transformation of *F. esculentum* has not yet been developed. In this study, we established an Agrobacterium-mediated transformation protocol for *F. esculentum* by adapting *an F. tataricum* procedure, incorporating a heat-shock treatment at 43 °C for 5 min. This achieved a transgenic frequency of 27.27% among regenerated plants (6/22) and an absolute editing efficiency of 4.55% (1/22) in the agronomically relevant ‘Panda’ cultivar. Targeted knockout of *FeS-ELF3 via* CRISPR/Cas9 yielded a self-compatible line with long-homostylous flowers. Plants derived from this single edited founder event exhibited architectural shifts - specifically reduced height and shorter inflorescences – without an apparent reduction in seed yield under greenhouse conditions. Because these observations stem from a single edited line, further evaluation of independent mutant lines or complementation assays is required to definitively distinguish *FeS-ELF3*-specific pleiotropic effects. Nevertheless, this study provides a proof-of-concept for overcoming self-incompatibility and establishes a genomic framework for the rapid improvement of *Fagopyrum esculentum*.

## 1 Introduction

Buckwheat (*Fagopyrum* spp.), a member of the Polygonaceae family, comprises 26 recognised species, among which *Fagopyrum esculentum* Moench (commonly referred to as sweet or common buckwheat) and *Fagopyrum tataricum* (L.) Gaertn. (known as bitter or Tartary buckwheat) are the most widely cultivated. As an underutilised orphan crop, buckwheat has considerable potential to enhance global food and nutritional security. Naturally gluten-free and rich in high-quality proteins, essential minerals, and bioactive compounds such as rutin, buckwheat is considered a functional food. Its seeds are typically dehulled and milled into flour, which serves as a base for food products such as bread, noodles, pasta, cakes, biscuits, and fermented products such as wine, beer, and vinegar. The flowers are highly valued for honey production due to their distinctive colour and abundant nectar. Importantly, buckwheat is a resilient crop that can thrive in low-fertility, acidic soils and high altitudes, where many staple cereals fail. This adaptability makes it particularly suitable for cultivation in marginal environments and is relevant to sustainable agriculture (Jha et al., 2024; Ding et al., 2025; Pinski et al., 2025). Despite its nutritional and ecological value, common buckwheat suffers from self-incompatibility and relies on insect pollinators for fertilisation, leading to unstable yields (Kasajima et al., 2017).

Successful fertilisation in *F. esculentum* occurs only between plants bearing complementary floral morphs - Thrum and Pin - due to a heteromorphic self-incompatibility (SI) system. This mechanism is genetically controlled by a hemizygous S-locus present in Thrum plants and is tightly linked to floral morphology. Previous studies have demonstrated that breakdown of SI and the emergence of long-homostyle flowers result from inactivation of *S-ELF3*, a homologue of *Arabidopsis thaliana EARLY FLOWERING3 (ELF3)*, located within the S-locus (Yasui et al., 2012). Mechanistically, *ELF3* acts as a central node in the plant circadian clock - via both Evening Complex-dependent and independent signalling - to coordinate photoperiodic flowering, architecture, and environmental adaptability under changing climate conditions. Consequently, targeted manipulation of *ELF3* offers a valuable strategy for crop optimisation. Nevertheless, while its role is well defined in *A. thaliana*, significant knowledge gaps remain in non-model crops such as Fagopyrum, where the phenotypic effects of *ELF3* disruption have yet to be systematically characterised (Zhu and Wang, 2024). In buckwheat, research on S-ELF3 has primarily focussed on its central role in floral morphology and SI. For instance, the *F. esculentum* PL4 line carries a nonsense mutation in *S-ELF3*, inherited from *F. homotropicum*. Additionally, ethyl methanesulfonate mutagenesis has produced a long-homostyle *F. esculentum* line due to splice site mutations in *S-ELF3.* This work relies on the analysis of single mutant alleles to establish gene–trait relationships at the S-locus (Fawcett et al., 2023). *F. tataricum*, a homomorphic and self-compatible species, exhibits structural variation in S-ELF3: an inverted duplication of the 5′ region and a retrotransposon insertion (Yasui et al., 2012). Collectively, these findings firmly establish *S-ELF3* as the key genetic determinant of SI in buckwheat. To overcome self-incompatibility in an elite genetic background, we established an Agrobacterium-mediated transformation protocol and targeted FeS-ELF3 with CRISPR/Cas9, thereby generating a self-compatible *F. esculentum* cv. ‘Panda’.

## 2 Results and discussion

### 2.1 Inactivation of the *FeS-ELF3* gene leads to self-compatible *F. esculentum* plants

The sequencing of the coding sequence of *FeS-ELF3* revealed no single-nucleotide polymorphisms between *F. esculentum* “Pintian4” and *F. esculentum* cv. ‘Panda’ (**Supplementary Data 1**). Given that *FeS-ELF3* is located within a hemizygous genomic region exclusive to Thrum plants (Fawcett et al., 2023), a Cas9 construct targeting *FeS-ELF3* was introduced into Thrum embryogenic calli induced from immature embryos using the Agrobacterium-mediated transformation system described here (**Supplementary Figures S1a, b**; **Supplementary Data 1**). The previously reported transformation protocol for *F. tataricum* was modified by the inclusion of a heat-shock step (43 °C for 5 min), which has been shown to increase transformation efficiency (Patel et al., 2013). Transgenic status was confirmed by PCR targeting a partial Cas9 fragment, yielding a frequency of transgenics among regenerated plants of 27.27% (6 transformed out of 22 regenerated plants) and an absolute editing efficiency of 4.55% (1 edited plant out of 22 regenerated plants). These results align with the ~20% transgenic frequency previously achieved using our optimised *F. tataricum* protocol (**Supplementary Figure S1c**; **Table 1**). However, while transformation efficiency remained relatively high, the regeneration rate for *F. esculentum* is notably low; consequently, multiple independent transformation cycles were required to produce viable plants. To overcome this limitation, vector constructs could be further optimised by incorporating developmental regulators, such as the GRF4–GIF1 chimaera, which are known to significantly enhance shoot regeneration efficiency in recalcitrant transgenic plants (Debernardi et al., 2020).

**Table 1:** Summary of *Agrobacterium tumefaciens* GV3101-mediated transformation and CRISPR/Cas9 editing efficiency in *Fagopyrum esculentum*.

Species	Target gene	Regenerated plants (T0​)	Frequency of transgenics among regenerated plants (T-DNA+)	Absolute editing efficiency
*F. esculentum*	*FeS-ELF3*	22	6/22 (27.27%)	1/22 (4.55%)

The frequency of transgenic regenerated plants is calculated as the percentage of regenerated plants containing T-DNA (T-DNA+). Absolute editing efficiency is calculated as the percentage of edited plants among all regenerated plants.

The targeted mutagenesis yielded a mutant carrying a single-nucleotide insertion in *FeS-ELF3*, resulting in a frameshift and the introduction of a premature stop codon, generating a loss-of-function allele (**Figure 1a**; **Supplementary Figure S2a**; **Supplementary Data 1**). The resulting *fes-elf3* mutant displayed long-homostylous flowers, a characteristic phenotype associated with the breakdown of self-incompatibility in *F. esculentum* (**Figure 1b**). T1 progeny were resequenced to confirm the inheritance of the targeted *FeS-ELF3* mutations identified in the parental T0 line (**Supplementary Figure S2b**). Subsequently, transgene-free T2 individuals were identified and characterised under greenhouse conditions. This stable transmission of edited alleles to the T1 generation demonstrates the effectiveness of this strategy for developing new buckwheat varieties.

**Figure 1:**
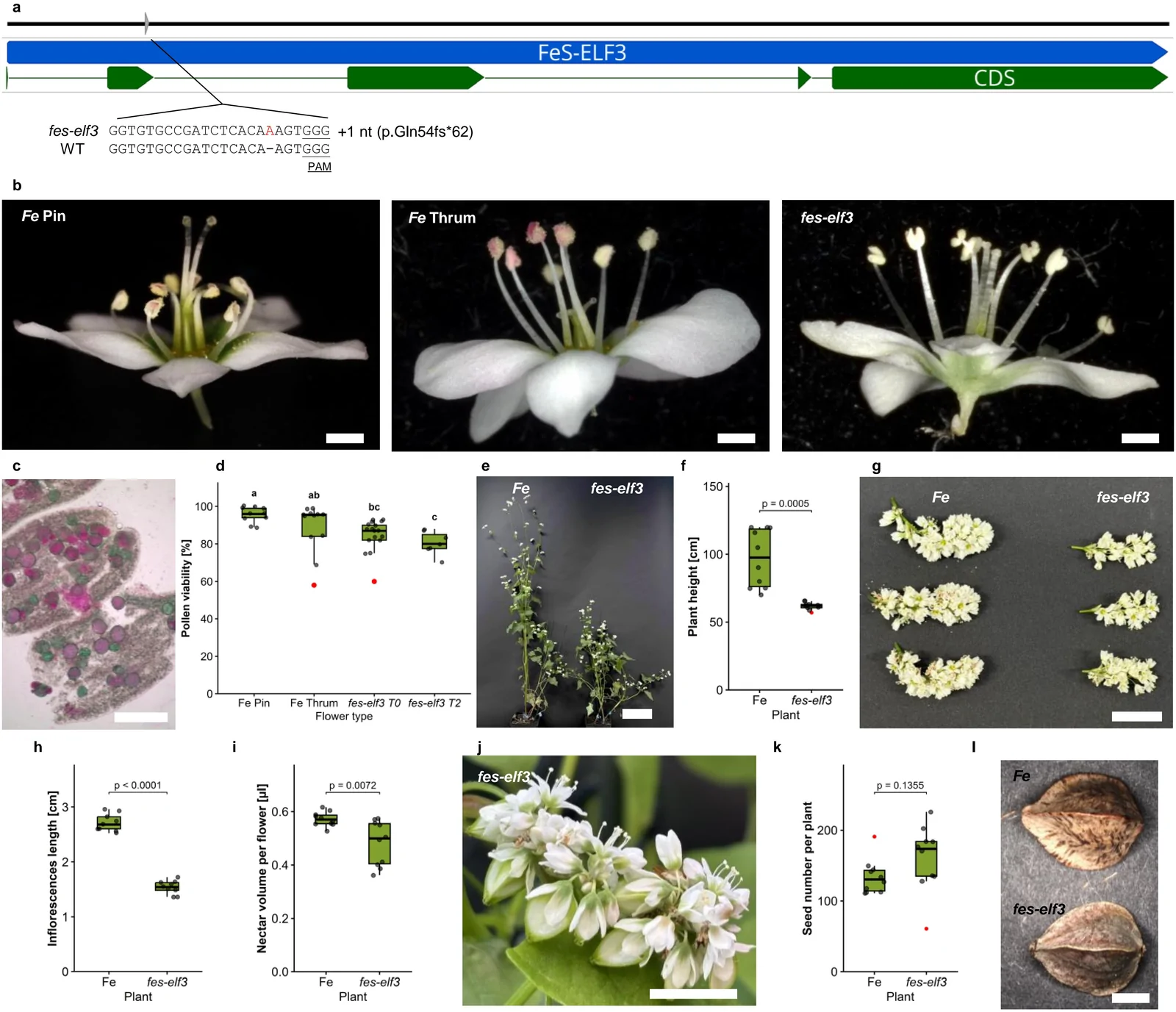
Inactivation of *FeS-ELF3* leads to long-homostylous flowers and self-compatibility. **(a)** Schematic representation of the *FeS-ELF3* gene located in a hemizygous genomic region specific to Thrum plants, with the sgRNA target site indicated. PAM, protospacer adjacent motif. **(b)** Floral morphotypes of *F. esculentum* cv. ‘Panda’. Pin flowers exhibit long styles and low anthers, whereas Thrum flowers have short styles and elevated anthers. Long-homostylous flowers result from nonsense mutations in *FeS-ELF3* from the T0 generation. Scale bar, 1 mm. **(c)** Pollen viability assessed by Alexander’s staining (Pin: n = 10; Thrum: n = 12; *fes-elf3* T0: n = 18; *fes-elf3* T2: n = 7). Scale bar, 100 µm. **(d)** Quantification of pollen viability in Pin, Thrum, and *fes-elf3* (T0 and T2 generations) flowers. **(e)** Representative photographs of *fes-elf3* (T2 generation) and WT plants grown under greenhouse conditions. Scale bar, 10 cm. **(f)** Plant height of *fes-elf3* (T2 generation) and WT plants grown in the greenhouse (n = 10 plants per genotype). **(g)** Representative inflorescences of *fes-elf3* (T2 generation) and WT plants. Scale bar, 2 mm. **(h)** Inflorescence length of *fes-elf3* (T2 generation) and WT plants (n = 9 plants per genotype, averaged from 5 inflorescences per plant). **(i)** Nectar volume per flower in *fes-elf3* (T2 generation) and WT plants. Nectar was collected from 50 flowers per plant and expressed as mean nectar volume per flower; n = 10 plants per genotype. **(j)** Inflorescence of *fes-elf3* (T2 generation) showing flowers and developing seeds. Scale bar, 1 cm. **(k)** Seed number per plant in *fes-elf3* (T2 generation) and WT under greenhouse conditions (n = 10 plants per genotype). **(l)** Representative seeds of *fes-elf3* (T2 generation) and WT plants. Scale bar, 2 mm. Boxplots indicate the median (horizontal line) and interquartile range (IQR; 25th–75th percentiles), with whiskers extending to 1.5×IQR. Data points beyond the whiskers were identified as outliers and are highlighted in red. Statistical significance was determined by Student’s *t*-test **(h, k)**, Welch’s t-test **(f, i)**, or Dunn’s test following Kruskal–Wallis **(d)**. Shared letters indicate no statistically significant difference (p > 0.05) **(d)**.

Reproductive potential was assessed by evaluating pollen viability in the T0 and T2 plants. Although pollen viability in *fes-elf3* was slightly reduced relative to Thrum and Pin flowers, (Thrum, 88.9 ± 13.1%; Pin, 95.6 ± 4.1%; *fes-elf3* T0, 83.4 ± 10.1%; fes-elf3 T2 80.4 ± 6.2%; mean ± SD; **Figures 1c, d**) (Kruskal-Wallis, H = 19.19, df = 3, p = 0.0002) mutant plants retained sufficient reproductive capacity to produce seeds under the tested conditions. Dunn’s *post hoc* test revealed that viability was significantly reduced in *fes-elf3* T0 (p_adjusted_ = 0.0021) and T2 (p_adj_ = 0.0014) compared to Pin flowers, as well as in T2 compared to Thrum flowers (p_adj_ = 0.0440), whereas all other pairwise comparisons were non-significant (p_adj_ > 0.05; **Figure 1d**). Mild reductions in pollen viability without loss of overall fertility align with findings in related early flowering (ELF) family knockouts; CRISPR/Cas9-edited *elf* mutants in *A. thaliana* exhibit impaired pollen germination and altered anther morphology relative to WT while retaining partial reproductive competence (Jan et al., 2026).

Furthermore, we observed that *fes-elf3* plants were significantly shorter than wild-type (WT) plants, with average heights of 61.9 ± 2.7 cm compared with 97.1 ± 21.1 cm in WT (Welch’s t-test, t(9.3) = 5.22, p = 0.0005) (**Figures 1e, f**). Phenotypic variance was also significantly lower in *fes-elf3* than in WT, as demonstrated by Levene’s test (F(1, 18) = 61.37, p < 0.0001). Consistent with this result, the coefficient of variation was substantially lower in *fes-elf3* (CV = 4.41%) than in WT (CV = 21.79%), indicating reduced phenotypic dispersion and greater uniformity in plant height among mutant individuals. However, because *F. esculentum* is an obligate outcrossing species, part of the reduced variance in the mutant line likely stems from its reduced genetic heterogeneity (as derived from a single homozygous line) compared to the genetically diverse wild-type population. This height reduction aligns with phenotypic effects observed in other crops; for example, in chickpea (*Cicer arietinum*), *ELF3* loss-of-function mutations confer photoperiod insensitivity - dramatically accelerating flowering under non-inductive short days to match long-day timing - which prematurely ends vegetative growth and yields significantly shorter, compact plants (Ridge et al., 2017). From an agronomic perspective, this combination of dwarfing and height uniformity in *fes-elf3* is highly desirable for improving lodging resistance and facilitating mechanical harvesting, aligning with ELF3’s established role in regulating shade avoidance and stem elongation (Jiménez-Gómez et al., 2010; Xiang et al., 2019; Hornyák et al., 2020). Indeed, a previously developed ethyl methanesulfonate–induced dwarf mutant of *F. tataricum* (*ftdm*) was shown to be suitable for high-density planting and commercial cultivation (Sun et al., 2021). In addition to reduced plant height, *fes-elf3* plants exhibited shorter inflorescences (1.54 ± 0.12 cm) relative to WT plants (2.71 ± 0.16 cm; **Figures 1g, h**). Variances did not differ significantly between the two groups (Levene’s test, *F*(1, 16) = 0.750, *p* = 0.3992), and a Student’s *t*-test confirmed a significant difference in inflorescence length (*t*(16) = 17.35, *p* < 0.0001).

To date, pleiotropic alterations in plant height or inflorescence architecture have not been reported for *fes-elf3* mutants, as *FeS-ELF3* has been characterised predominantly as a specific regulator of style length and female compatibility (Ma et al., 2022; Fawcett et al., 2023; He et al., 2023; Lin et al., 2023). However, given its function as a transcriptional regulator, *FeS-ELF3* likely modulates downstream gene networks involved in vegetative elongation and inflorescence development (Yasui et al., 2012; Zhu and Wang, 2024). Alternatively, these unexpected phenotypic shifts could result from somaclonal variation induced during tissue culture or Cas9 off-target activity. To evaluate specificity, we analysed six predicted off-target sites via Sanger sequencing; no off-target mutations were detected (**Supplementary Data 1**), suggesting a low likelihood of detectable off-target editing at these loci. All predicted genic off-target sites were experimentally tested, whereas only intergenic sites remained untested. Furthermore, *FeS-ELF3* knockout renders the plants self-compatible, which facilitates stable line propagation, but continuous selfing inherently reduces genetic diversity across generations (Peer and Mir, 2025). Evaluating additional independent lines and performing transgenic complementation will further rule out tissue culture effects and confirm generalisability.

Given that *F. esculentum* is an important nectar source for pollinators (Plazek et al., 2023), nectar production was also evaluated. Nectar volume per flower was moderately reduced in *fes-elf3* plants (0.481 ± 0.083 µL) compared with WT plants (0.572 ± 0.027 µL; **Figure 1i**). Homogeneity of variance was violated (Levene’s test, F(1, 18) = 11.517, p = 0.0032); therefore, Welch’s t-test was used and revealed a significant difference in nectar volume between WT and *fes-elf3* plants (t(10.9) = 3.30, p = 0.0072). Reduced nectar production in *fes-elf3* plants could potentially decrease the floral reward available to pollinators, as reduced nectar availability in buckwheat during drought stress has been associated with lower pollinator attraction and cascading effects on reproductive success (Rering et al., 2020). Although the potential consequences for seed production may be partly mitigated by the self-compatibility of *fes-elf3* plants, the reduction in nectar availability may still influence plant-pollinator interactions and warrants further investigation.

To verify autonomous self-compatibility, *fes-elf3* plants were isolated in a separate growth chamber devoid of insect pollinators or mechanical cross-pollination. Under these isolated conditions, the mutant plants successfully set seeds, providing direct functional evidence of self-compatibility (**Figure 1j**). Seed number per plant was comparable between WT (134.2 ± 24.6) and *fes-elf3* (160.3 ± 46.8) plants under greenhouse conditions (**Figures 1k, l**). Variances did not differ significantly between the two groups (Levene’s test, F(1, 18) = 1.926, p = 0.1821), and a Student’s t-test indicated no significant difference in seed number (t(18) = −1.56, p = 0.1355). It should be emphasised that these experimental environments differed in design: obligate self-incompatible WT plants were maintained with *Calliphora vicina* flies to facilitate necessary cross-pollination, whereas self-compatible *fes-elf3* mutants were strictly isolated without insect vectors to verify autonomous selfing capacity. While this isolation confirms that *fes-elf3* can set seeds without pollinator assistance, comparing direct yield potential between these distinct microenvironments requires caution. Large-scale field trials in a uniform environment will be essential for precisely quantifying the commercial yield capacity of *fes-elf3* mutant. Given previous results of stable high-yielding self-compatible *F. esculentum* (Kasajima et al., 2017), we hypothesised that self-compatible lines of *F. esculentum* cv. ‘Panda’ could offer more consistent year-to-year yields by reducing reliance on pollinator availability. In self-incompatible buckwheat, pollination success is vulnerable to unpredictable weather conditions, particularly given the narrow one-day flowering window of individual blossoms (Cawoy et al., 2009). However, while our greenhouse observations demonstrate viable seed production in self-compatible plants, validating these potential agronomic benefits will require extensive multi-year, multi-location field trials across diverse climatic conditions.

In summary, this study demonstrates the efficacy of CRISPR/Cas9-mediated genome editing to overcome heteromorphic self-incompatibility in common buckwheat. We successfully generated a self-compatible line in the commercially relevant Polish cultivar ‘Panda’ and confirmed the stable inheritance of the edited trait in the T1 generation, and conducted phenotypic characterisation using transgene-free T2 plants. A current limitation of this study is that only a single *fes-elf3* mutant line was obtained, warranting further independent transformation events to fully validate phenotypic consistency and to advance mechanistic understanding of *FeS-ELF3*. Nonetheless, as a New Genomic Technique (NGT) with a globally favourable regulatory trajectory compared to traditional GMOs (Menary and Fuller, 2024), this self-compatible ‘Panda’ line represents a valuable genetic resource. It can be integrated directly into agricultural production or leveraged in breeding programs (Suzuki et al., 2014) to stack traits such as dwarfism, waxy starch, reduced allergenicity, and abiotic stress tolerance, ultimately contributing to resilient food systems.

## 3 Methods

### 3.1 Plant material

Embryogenic calli of *F. esculentum*, derived from Thrum immature zygotic embryos, were used for Agrobacterium-mediated transformation. Because *FeS-ELF3* is localised within a hemizygous genomic region unique to the S-locus of Thrum individuals (Fawcett et al., 2023), verification of its presence confirms the Thrum origin of the tissue. Once sufficient tissue was obtained, genomic DNA was isolated using the CTAB method. A fragment of the *FeS-ELF3* gene was subsequently amplified (FeS-ELF3_partial_F and FeS-ELF3_partial_R) using Q5 High-Fidelity DNA Polymerase (NEB, UK) and sequenced to verify the Thrum-type origin of the callus and confirm the presence of the *FeS-ELF3* gene (**Supplementary Figure S1a**, **Supplementary Data 1**). The oligonucleotides used in this study are listed in **Supplementary Data 2**. The callus was maintained on RX medium according to (Betekhtin et al., 2017) at 25 °C (± 1 °C) in the dark and subcultured every two weeks. Before transformation, embryogenic Thrum type callus of *F. esculentum* was put on regeneration medium containing macro- and micro-elements as in MS medium (Murashige and Skoog, 1962), 3.0 mg/L 6-benzylaminopurine, 1.0 mg/L thidiazuron, 30 g/L sucrose, 3 g/L phytagel and cultured in a growth room at 28°C (± 1 °C) with a 16/8 h (light/dark) photoperiod, under light intensity of 55 µmol/m^2^/s for 12–14 days to induce somatic embryos. Callus with forming somatic embryos was transformed.

### 3.2 Cloning of *FeS-ELF3*, vector design, and selection of CRISPR/Cas9 target sites

To validate the *FeS-ELF3* coding sequence in *F. esculentum* cv. ‘Panda’ for sgRNA design, total RNA was isolated from young Thrum-type flowers using the RNeasy Mini Kit (Qiagen, Hilden, Germany). First-strand cDNA was synthesised using the Maxima H Minus First Strand cDNA Synthesis Kit with dsDNase (Thermo Scientific, Waltham, MA, USA). The complete *FeS-ELF3* coding sequence was subsequently amplified using Q5 High-Fidelity DNA Polymerase (NEB, UK) with the primers BsaI_FeS-ELF3_F and BsaI_FeS-ELF3_R. These oligonucleotides were designed using the *F. esculentum* “Pintian4” reference genome from the Chinese National Genomics Data Center (GWHBJBK00000000). The oligonucleotides were designed to remove the STOP codon and add BsaI recognition overhangs, allowing Golden Gate cloning into vector pGGC000 from the GreenGate cloning system. The vectors with cloned *FeS-ELF3* were sequenced using M13F and SP6 oligonucleotides (**Supplementary Data 1**). For targeted mutagenesis, we used the binary vector pGG-Cas9-AtU6-lacZ-kanR, constructed using the GreenGate cloning system (Lampropoulos et al., 2013). The empty vector was assembled as described in Pinski and Betekhtin (2023) with two modifications: pGG-Cas9(ΔSTOP) was replaced with pGG-Cas9(STOP), and the pGGD001 (linker-GFP) module was substituted with pGGD002 (C-tag, D-dummy) (**Supplementary Figure S3**, GenBank accession number: PZ366425). sgRNA sequences targeting *FeS-ELF3* were designed using Geneious Prime 2022.0.2. sgRNA specificity was assessed with Cas-OFFinder (Bae et al., 2014) against the *F. esculentu*m ‘Pintian4’ reference genome, allowing up to five mismatches and no insertions or deletions. The selected sgRNA sequence was 5′-GGTGTGCCGATCTCACAAGT-3′ (PAM: 5′-GGG-3′). Final sgRNA selection was based on predicted editing efficiency, proximity to the 5′ end of the coding region, and the fewest off-target sites in the reference genome. Target-specific sgRNA sequence was introduced into the vector pGG-Cas9-AtU6-lacZ-kanR via Golden Gate assembly using flanking AarI restriction sites, and the assembly was confirmed by sequencing. The assembled vector was electroporated into the *A. tumefaciens* GV3101 strain.

### 3.3 Agrobacterium-mediated transformation

Agrobacterium preparation, callus transformation, and plant regeneration were performed according to the protocol previously described by Pinski and Betekhtin (2023) for Agrobacterium-mediated transformation of *F. tataricum*, with minor modifications. For *F. esculentum*, Thrum type callus with somatic embryos (5–7 g) was placed in 30 mL of liquid RX medium supplemented with 30 mg/L acetosyringone and 0.1% Pluronic F-68 for heat shock in a water bath at 43 °C for 5 min. Afterwards, the RX liquid medium was removed, and the tissue was incubated in 30 mL of Agrobacterium suspension for 10 min. Subsequently, the Agrobacterium suspension was removed, and the tissue was dried on 110 mm filter papers in open Petri dishes for 15 min. The next steps were the same as for *F. tataricum* Agro-transformation. To minimise the occurrence of chimeric plants, the kanamycin concentration used for selection was increased from 50 mg/L to 100 mg/L, and the selection period was extended from 3–4 weeks to 6–7 weeks.

### 3.4 Regenerated plants cultivation

Regenerated plants with developed roots were transferred to an aero-hydroponic propagator for 20 cuttings (Hemp) filled with medium containing 20 ml of Aqua Vega A (Canna, Netherlands) and 20 ml of Aqua Vega B (Canna, Netherlands) per 10 L of water. For the first week, the lid was applied to maintain humidity; after this time, the lid was gradually removed to acclimate the plants (**Supplementary Figure S1b**). Rooted and strong plants were transferred to pots filled with moss-coconut fibre substrate (Ceres International) and grown under greenhouse conditions (18–26 °C, 16/8 photoperiod, light intensity of 40 µmol/m^2^/s sodium lamps Lucalox LU600W/PSL, HU, optimally irrigated). Seeds from the T0 generation were collected in successive rounds. The *fes-elf3* plants were maintained in a greenhouse chamber without any other *F. esculentum* plants to prevent cross-pollination. The nectar analysis was performed as described previously (Plazek et al., 2023); nectar was collected from 50 open flowers per plant, and the total nectar volume was divided by the number of flowers to calculate nectar volume per flower. Ten plants were analysed per genotype, and each plant was treated as an independent biological replicate. Wild-type *F. esculentum* plants were grown in a separate chamber containing *Calliphora vicina* flies, enabling efficient cross-pollination between Pin and Thrum plants.

### 3.5 Mutants characterisation

The DNA from the leaves of regenerated plants was isolated using the CTAB method, as previously described by Hus et al (Hus et al., 2020). Successful transformation and T-DNA integration were first confirmed by PCR amplification of a fragment of the Cas9 gene (Cas9_partial_F and Cas9_partial_R). In the case of *FeS-ELF3*, because a single copy of the gene is present in *F. esculentum* Thrum plants, the PCR product was directly sequenced. The resulting sequencing data were aligned with the corresponding reference sequences using Geneious Prime 2022.0.2 to identify potential mutations within the target genes and to characterise potential influence on protein formation. The Sanger sequencing results for WT and mutant sequences are in **Supplementary Data 1**. The transgene-free plants were assessed by PCR amplification of a fragment of the Cas9 gene (Cas9_partial_F and Cas9_partial_R). Among the 24 potential off-target sites identified, six were prioritised - favouring those within gene coding regions - and validated via Sanger sequencing (**Supplementary Table 1**) using the primers given in **Supplementary Data 2**. Of these, five were located within gene bodies, including two in exonic regions and three in intronic regions, while one site was located in an intergenic region. Sanger sequencing trace files were subsequently analysed for indel frequencies using Tracking of Indels by Decomposition (TIDE) (Brinkman et al., 2014) to estimate indel proportions and size distributions. The alignment window was set to 100 bp upstream of the predicted Cas9 cleavage site, and the decomposition window extended 100 bp downstream of the cleavage site. Indels ranging from 1 to 20 bp were considered, with a significance cut off for decomposition set at p < 0.001. All primary sequencing results are provided in Supplementary Data 1.

### 3.6 Microscopic observations

The bright field images of the flower and seeds were captured using a KEYENCE VHX-7000 digital microscope (Keyence, Japan).

### 3.7 Pollen viability

Pollen viability was assessed according to Alexander’s staining method (Alexander, 1969). Fully open flowers were collected from the greenhouse-grown plants (Pin: n = 10; Thrum: n = 12; *fes-elf3* T0: n = 18; *fes-elf3* T2: n = 7, where n represents individual flowers, each collected from a separate plant). Then, fresh pollen was placed on a drop of Alexander’s stain on microscope slides and covered with a coverslip. The slides were examined under an inverted Leica DMi8 microscope (Leica Microsystems, Germany) equipped with a Leica DFC 7000 T camera conjugated with LAS X Extended Depth of Field and Deconvolution Modules. The number of viable (dark red cytoplasm with a green wall) and non-viable (entirely green) pollen grains was counted. Pollen viability was expressed as the percentage of viable pollen.

### 3.8 Statistical analysis

Statistical analyses were performed in RStudio (R version 4.4.1; RStudio PBC, Boston, MA, USA) using the following R packages: readxl, dplyr, tidyr, purrr, rstatix, ggplot2, ggsignif, patchwork, and RColorBrewer. Unless otherwise indicated, data are presented as mean ± standard deviation (SD). The individual plant was the experimental unit for plant height (cm), inflorescence length (cm; n = 9 plants per genotype, averaged from 5 inflorescences per plant), nectar volume (µL per flower; n = 10 plants per genotype, averaged from 50 flowers per plant), and seed number per plant; pollen viability (%) was assessed per flower.

Data distributions were evaluated for normality using the Shapiro–Wilk test and inspection of Q–Q plots, and homogeneity of variances was assessed using Levene’s test. For two-group comparisons of normally distributed data with equal variances, two-sided Student’s t-tests were used (inflorescence length, seed number). When the assumption of homogeneity of variance was violated, Welch’s t-test was applied (plant height, nectar volume). For multi-group comparisons of non-parametric data (pollen viability), the Kruskal–Wallis test was conducted, followed by Dunn’s *post hoc* pairwise test with Bonferroni correction for multiple testing. Statistical significance was defined as p < 0.05 or p_adj_ < 0.05 for pairwise comparisons. Potential outliers were identified using boxplots based on the 1.5×IQR criterion. All observations were retained in the statistical analyses, and no data points were excluded.

## Data Availability

The datasets presented in this study can be found in online repositories. The names of the repository/repositories and accession number(s) can be found in the article/**Supplementary Material**.

## Supplementary material

Supplementary Figure 1 Agrobacterium-mediated transformation of *F. esculentum*. **(a)** Callus derived from *F. esculentum* Thrum type. Scale bar, 2 mm. **(b)** Regenerated *F. esculentum* plant. **(c)** PCR identification of transformed plants with integrated T-DNA (by detection of a fragment of the *Cas9* gene using oligonucleotides Cas9_partial_F and Cas9_partial_R). Plant number 20 carries a mutated *fes-elf3* copy, which was further characterised in this study.

Supplementary Figure 2 Sequence alignment of *fes-elf3* mutants. **(a)** Sequence profile of the *fes-elf3* T0 founder line. **(b)** Representative sequences of ten T1 progeny derived from the *fes-elf3* T0 mutant, demonstrating the stable inheritance of the edited alleles. The WT reference, sgRNA target site, and PAM sequence are indicated.

Supplementary Figure 3 Plasmid map of pGG-Cas9-AtU6-lacZ-kanR, a binary CRISPR/Cas9 vector for sgRNA-mediated targeted mutagenesis in buckwheat. The T-DNA region, flanked by left (LB) and right (RB) borders, contains the AtU6–26 promoter driving sgRNA expression, with the protospacer cloning site defined by flanking AarI restriction sites and the gRNA scaffold. Cas9 is expressed from the CaMV 35S promoter, and the kanamycin plant selectable marker (neomycin phosphotransferase II) from the pNOS promoter. The bacterial backbone carries a spectinomycin resistance cassette (Sp^R^) for bacterial selection, a pBR322 origin, and the pVS1 replicon for stable maintenance in *Agrobacterium tumefaciens*.

Supplementary Data Sheet 1 The Sanger sequencing results of WT sequences and mutants of *F. esculentum*.

Supplementary Data Sheet 2 Oligonucleotides used in the study. Regions of sgRNA sequence overlap are underlined. Lowercase letters indicate cloning overhangs. A bold red “G” highlights the C->G substitution introduced to eliminate a stop codon.

Supplementary Table 1 Potential off-target sites identified by Cas-OFFinder with parameters allowed up to five mismatches with no bulges.
